# A review of changes to the attention deficit/hyperactivity disorder age of onset criterion using the checklist for modifying disease definitions

**DOI:** 10.1186/s12888-019-2337-7

**Published:** 2019-11-12

**Authors:** Sharon Sanders, Rae Thomas, Paul Glasziou, Jenny Doust

**Affiliations:** Institute for Evidence-Based Healthcare, Faculty of Health Sciences and Medicine, Bond University, Level 4, Building 5, Gold Coast, Queensland 4226 Australia

**Keywords:** Attention deficit/hyperactivity disorder, Disease definitions, Overdiagnosis

## Abstract

**Background:**

Widening definitions of health conditions have the potential to affect millions of people and should only occur when there is strong evidence of benefit. In the last version of the Diagnostic and Statistical Manual of Mental Disorders (DSM), the DSM-5 Committee changed the Attention Deficit Hyperactivity Disorder (ADHD) age of onset criterion in two ways: raising the age of symptom onset and removing the requirement for symptoms to cause impairment. Given concerns about ADHD prevalence and treatment rates, we aimed to evaluate the evidence available to support these changes using a recently developed Checklist for Modifying Disease Definitions.

**Methods:**

We identified and analysed research informing changes to the DSM-IV-TR ADHD age of onset criterion. We compared this evidence to the evidence recommended in the Checklist for Modifying Disease Definitions.

**Results:**

The changes to the DSM-IV-TR age of onset criterion were based on a literature review (publicly available as a 2 page document with online table of included studies), which we appraised as at high risk of bias. Estimates of the change in ADHD prevalence resulting from change to the age of onset criterion were based on a single study that included only a small number of children with ADHD (*n* = 68) and only assessed the impact of change to the age component of the criterion. No evidence was used by, or available to the Committee regarding the impact on prevalence of removal of the requirement for impairment, or the effect of the criterion changes on diagnostic precision, the prognosis of, or the potential benefits or harms for individuals diagnosed by the new, but not old criterion.

**Conclusions:**

The changes to the age of onset criterion were based on minimal research evidence that suffered from either high risk of bias or poor applicability. The minimal documentation available makes it difficult to judge the rigor of the process behind the criterion changes. Use of the Checklist for Modifying Disease Definitions would assist future proposed modifications of the DSM ADHD criteria, provide guidance on the studies needed to inform potential changes and would improve the transparency and documentation of the process.

## Background

When the Diagnostic and Statistical Manual of Mental Disorders (DSM-IV-TR) became DSM-5, the age of onset criterion for Attention Deficit Hyperactivity Disorder (ADHD) changed from, ‘*some hyperactive-impulsive or inattentive symptoms that caused impairment were present before age 7 years*’ (DSM IV-TR), to ‘*several inattentive or hyperactive-impulsive symptoms present prior to age 12 years*’ (DSM-5). The modification thus comprised two changes: increasing the age of onset of symptoms from before 7 years to before 12 years, and removing the requirement for the “*onset of symptoms causing impairment”* to the “*onset of symptoms*”. Both changes widen the definition of ADHD and potentially lead to the widening of treatment recommendations. The increase in the prescribing of ADHD medications in several countries in recent years is a possible consequence of this widening, raising concerns about overdiagnosis [1–5].

In response to the widening of definitions of health conditions observed across many disciplines [6], a checklist and guide for those considering changes to disease definitions has recently been published [7]. Rigorously developed by a multidisciplinary, multicontinent author group for the Guidelines International Network Preventing Overdiagnosis working Group members, the Checklist for Modifying Disease Definitions, provides a framework of 8 items to guide the decision-making process regarding the uncertainties and trade-offs in modifying disease definitions (Table 1). Five checklist items require the identification and analysis of research studies to determine: potential changes in prevalence, the prognostic ability, precision and accuracy, and the incremental benefits and harms of the new definition.

**Table 1:** Checklist for Modifying Disease Definitions [7]

Checklist Item	Rationale
1. Definition: What are the differences between the previous and the new definition?	It is important to delineate the proposed change precisely.
2. Number of people affected: How will the new disease definition change the incidence and prevalence of the disease?	The number of people affected is extremely important in understanding benefits, harms and resources needed.
3. Trigger: What is the trigger for considering the modification of the disease definition?	Stating the trigger for considering modification helps understand the necessity for modifying the disease definition.
4. Prognostic ability: How well does the new definition of disease predict clinically important outcomes compared with the previous definition?	The most important feature of a disease definition is its ability to accurately predict clinically important outcomes.
5. Disease definition precision and accuracy: What is the repeatability, reproducibility and accuracy (when estimations are possible) of the new disease definition?	Disease definitions that are repeatable and reproducible improve the consistency of clinical decision making. Accuracy is often not able to be estimated because of the lack of a reference standard.
6. Benefit: What is the incremental benefit for patients classified by the new definition versus the previous definition?	Benefits of the disease definition can be outlined, using methods such as GRADE. It is particularly important to estimate benefits in conditions where the new definition will be used to determine treatment thresholds.
7. Harm: What is the incremental harm for patients classified by the new definition versus the previous definition?	Harms may also be outlined using methods such as GRADE. It is often more difficult to quantify harms, and particularly the psychosocial harms and harms on the societal level, including resource related harms.
8. Net benefit and harms: What is the net benefit and harm for patients classified by the new definition versus the previous definition?	A panel should consider all the above, and the balance of net benefits and harms prior to modifying a disease definition.

The development of the checklist allows a rigorous appraisal of the methods used when professional groups change the definition of a health condition. Given the concerns regarding the changes to the ADHD age of onset criterion, we used the Checklist for Modifying Disease Definitions, to examine changes to the diagnostic criteria for ADHD from DSM IV-TR to DSM-5. While the transition to DSM-5 involved changes to several of the ADHD criteria (e.g. the elaboration of symptom criterion and removal of exclusionary disorders criterion), we focus only on modification to the age of onset criterion. Our objectives were to; a) identify and appraise the research used by the relevant DSM-5 Committee to inform the changes to the age of onset criterion; and b) identify and evaluate any other research relating to the criterion as recommended by the checklist.

## Methods

### Identifying documents describing the proposed changes and supportive evidence

We first sought to identify documents outlining the proposed or actual changes to the DSM-IV-TR ADHD age of onset criterion and the evidence used by the Committee to inform these changes. We searched websites and bibliographic databases, asked manuscript authors’ and colleagues for studies known to them, and conducted reference checks, forward citation and PubMed “similar articles” searches (Additional file 1: Table S1).

### Identifying research to address checklist items

We then conducted a search for any further studies available in the literature that could address the checklist items requiring analysis of research studies (items 2, 4, 5, 6 and 7) and provide information to inform the proposed change to the age of onset criterion. We searched PubMed from 1990 to January 2013 using terms related to ADHD and age of onset (Additional file 1: Table S2). As DSM-5 was released in May 2013, we searched for studies that would have been available to the Committee up to and the end of January 2013. We performed reference checks and forward citations searches of relevant papers. Titles and abstracts were screened by two authors and potentially relevant studies were obtained in full text for further review.

### Classifying studies and analysing quality

Studies identified in both searches were independently assessed by two authors (SS, RT) and based on the study’s reported objectives (or first reported results if the objectives were unclear), categorized according to the checklist items as research relating to 1) prevalence, 2) prognosis, 3) accuracy and/or precision, and 4) benefits or harms. Studies not relating to these constructs were not further assessed. Studies that were not primary research studies (e.g. review articles), and studies concerned with different DSM versions or populations were also excluded from further assessment.

The quality of the studies addressing the checklist items was assessed using relevant risk of bias tools. We assessed the methodological quality of systematic reviews using A Measurement Tool to Assess Systematic Reviews (AMSTAR-2) [8]. For primary studies of prevalence, we used the risk of bias tool developed by Hoy [9]. For studies assessing the measurement properties of the age of onset criterion we used the QAREL appraisal tool [10] and for studies assessing the prognostic ability of the criterion we used the QUIPS tool [11]. To assess risk of bias in studies reporting treatment benefit and harm we used tools appropriate for the design of the study.

We assessed the strength of the research evidence for each Checklist item using elements from the Grading of Recommendations, Assessment, Development and Evaluations (GRADE) approach [12]. These include study design and limitations (e.g. risk of bias), the consistency of the research evidence (e.g. the similarity in magnitude and direction of results across studies of the same or similar design), and the applicability of the evidence to the Checklist question. To assess applicability we considered whether; prevalence studies evaluated the effect of the change in the age of onset criterion (including both age and impairment changes) on prevalence of ADHD; prognostic studies evaluated the clinically relevant outcomes of individuals diagnosed by the new, but not old age of onset criterion (i.e. the ‘additional’ children classified using the new criterion); studies of diagnostic precision evaluated the repeatability of the criterion by the same clinician at different times, or the reproducibility of different clinicians using the same or different measurement tools; and studies of treatment evaluated potential benefits and harms for those diagnosed by the new criterion and not diagnosed by the old criterion.

## Results

### Studies considered by the committee during revisions to the age of onset criterion

**Fig. 1:**
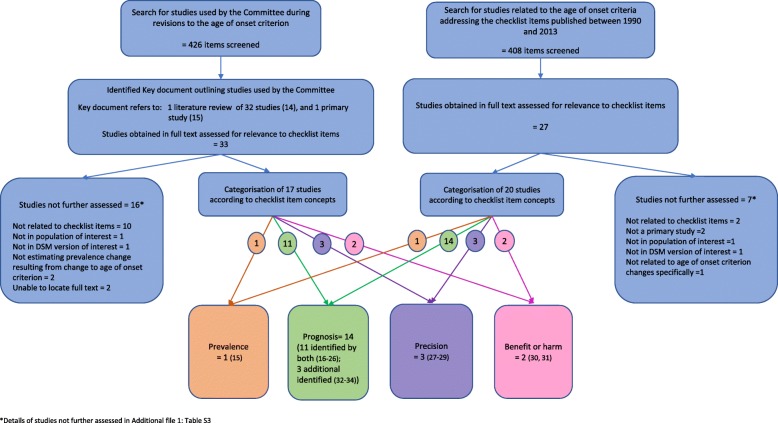
Categorisation of studies addressing checklist items and overlap with studies used by the Committee

We identified one document that we considered the key document describing the evidence to support the ADHD criteria revision process [13] (Additional file 1: Additional Document). The document refers to a systematic literature review published by the “workgroup age-of-onset subcommittee” [14] and one published study [15] as evidence for the change to the age of onset criterion. The review included 32 studies related to the age of onset criterion of varying designs and with different objectives. Based on these studies, the Committee commented on a) the magnitude of change (to the criterion), b) the reason/evidence for change, c) the potential negative consequences considered and d) additional objections and response. This key document had been available previously on the American Psychiatric Association (APA) website, but is no longer publicly available. On full text review of the 33 studies referred to by the key document, 17 studies addressing the checklist items were categorised and analysed (Fig. 1) [15–31].

### Studies relevant to the age of onset criterion and the checklist items published between 1990 and 2013

Our searches of the available literature found 20 relevant studies (Fig. 1) [15–34]. We did not locate any studies of prevalence, precision, benefit or harm additional to those used by the Committee. However, we identified a further 3 studies related to prognosis [32–34].

### Risk of bias and strength of evidence of the included studies

The published form of the literature review [14] referenced in the key document is a 2-page document. It provides a link to a supplementary online table that describes some features of the included studies (study objective, source of age of onset information, DSM version, study groups and results). We assessed the literature review as low quality according to the AMSTAR-2 quality assessment tool for systematic reviews [8] (Additional file 1: Table S4). The review did not meet 11 of the 13 applicable AMSTAR-2 items (of the 16 AMSTAR-2 items, 3 were not applicable as no meta-analysis was conducted): it did not specify a research question, outline apriori methods, explain how studies were selected and included, extract data in duplicate, list and justify excluded studies, describe included studies sufficiently, assess risk of bias of the included studies or account for risk of bias when interpreting the results or explain heterogeneity. In addition, funding sources for the included studies and the review authors’ potential conflict of interest were not reported (Item 10 and 16). The review partially met, or met, only two AMSTAR-2 criteria: the comprehensiveness of the search strategy and duplicate study selection. Flaws in the conduct or reporting of several critical domains of bias suggest that the review may not provide an accurate and comprehensive summary of the available studies.

### Analysis of studies assessing prevalence

The key document included a primary study assessing the effect of the change in the age of onset criterion on prevalence using a prospective cohort study of 2322 British twins assessed at 7 and 12 years of age using information from mothers and teachers [15]. At age 12, 66 children (3.3%) met the study criteria for ADHD (mother or teacher report of six or more inattentive and/or six or more hyperactivity-impulsivity symptoms) with age of onset of symptoms before 7 years. An additional 2 children presented with symptoms meeting the ADHD diagnostic criteria between age 7 and 12. This study estimated that the increase in the prevalence of ADHD because of the change in the criterion would be 0.1% (Additional file 1: Table S5a).

The research evidence related to the effect on prevalence used to support the change to the age of onset criterion consists of a single study. We assessed the risk of bias in this study as low (Additional file 1: Table S5b). However, there is considerable uncertainty about the applicability of the evidence because of the type of participants (twins), failure to assess the effects on prevalence of the change to both the age and impairment requirements of the criterion, the prospective measurement of symptom onset and the low prevalence of ADHD in the population studied (3.3%).

### Analysis of studies assessing prognosis

The key document considered the prognostic ability of the age of onset criterion using 11 studies [16–26] identified by the literature review [14] and we identified an additional 3 studies [32–34] in our searches (Fig. 1). These studies of cross-sectional and cohort design in child, adolescent or adult populations, compared a range of outcomes between variably defined groups with ‘early’ onset of ADHD symptoms or impairing symptoms (sometimes termed ‘Full ADHD’) and ‘late’ onset of ADHD symptoms or impairing symptoms (Additional file 1: Table S6a). Study results were mixed, with prognosis varying between ‘early’ and ‘late’ symptom onset groups for the same outcomes between studies, and for different outcomes within studies. Some of these studies were mentioned and referenced in the text of the literature review [14], while others appeared in the supplementary online table only.

The research evidence related to the prognostic ability of the age of onset criterion consists of multiple studies that we assessed to be at moderate or high risk of bias on 3 or more of the 6 domains of bias [11]. Risk of bias from confounding was present in most studies, and the potential for selective reporting or absence of an appropriate statistical model in all studies (Additional file 1: Table S6b). The possibility of bias arising from the retrospective recall of the age of symptom onset was judged to be considerable in the studies of cross-sectional design. In assessing the strength of the evidence, there was inconsistency in the results of studies of similar design, and the applicability of these studies is questionable as many were performed in clinical samples and none evaluated the prognostic ability of the criterion in individuals identified by the new but not old (DSM-IV-TR) age of onset criterion.

### Analysis of studies assessing precision

The key document considered 3 studies [27–29] included in the literature review [14] related to the precision of methods of measuring age of onset. These studies evaluated the reporting of the date, or the age of onset of symptoms or behaviours by the *same* informant (using the same or different method of obtaining onset information) at different time intervals (1 week, 1 year and 5 years) (Additional file 1: Table S7a). These studies found poor to moderate agreement on: the date of symptom onset when data were collected at interviews 1 week apart; but reasonable stability of mothers reporting DSM-III symptoms over a 1-year period; and parent or self-reports of ‘later’ age of onset of impairing symptoms after a 5-year interval.

The research evidence related to the precision that the Committee considered in changing the age of onset criterion was not applicable to the checklist item. The item requires evaluation of the agreement between the *same* clinicians at *different* times (repeatability), and between *different* clinicians (reproducibility) on their judgment of whether an individual meets, or does not meet, the *new* age of onset criterion. None of the available studies provide this information. In addition, we assessed the studies used by the Committee to be at high or unclear risk of bias because the study population was not suited to evaluate the precision of the age of onset criterion, the interviewers were not those who would perform the test in everyday practice, and because of the availability of other clinical information which may influence assessors coding of the date or age of onset. (Additional file 1: Table S7b).

### Analysis of studies assessing benefits and/or harms

The Committee considered 2 studies [30, 31] relating to the potential benefits and harms of treatment. The first of these single arm studies evaluated the response to methylphenidate among a population of adults with ‘late onset’ ADHD [30]. In the second, children, adolescents and adults were treated with methylphenidate and treatment response was compared between participants meeting DSM-IV ADHD criteria and participants meeting all criteria except symptom onset before age 7 [31] (Additional file 1: Table S8a). The adverse effects of the medication were reported in one study. There were no studies evaluating the potential harms arising from a diagnosis of ADHD according to the new age of onset criterion or arising from treatment of individuals classified by the new criterion.

To assess risk of bias in single-arm studies included in systematic reviews, we used a tool currently being trialed by the Cochrane Kidney Group (Beller, E. Personal communication. 2018. Feb 14). The research evidence related to the benefit or harm for individuals diagnosed by the new age of onset criterion consists of two single arm studies that are at high risk of bias due to lack of a control group, selection bias, lead time bias, bias due to adjunctive therapies, attrition bias and selective reporting of outcomes (Additional file 1: Table S8b). Further, the applicability of the studies to the checklist item is uncertain as the studies do not evaluate the benefits or harms arising from the treatment of those individuals identified by the new, but not old age of onset criterion (that is, the additional ‘milder’ and ‘older’ individuals identified by the new but not old criterion).

## Discussion

We used the Checklist for Modifying Disease Definitions as a framework to examine the research informing changes to the DSM-IV-TR ADHD age of onset criterion. We found that the research evidence used by the Committee to inform the change to the age of onset criterion was limited; often at high risk of bias and/or of limited applicability. No research was found to support the removal of the requirement for impairment component of the age of onset criterion. The process used to inform the changes to the criterion was not transparent, with only limited information publicly available and some of the previously available information no longer available. As outlined in the key document (Additional Document), the age of onset criterion included in the DSM-IV-TR has been criticized as being arbitrary, with evidence that the age 7 cut-off is not valid [35, 36]. This evidence would be considered in the checklist by the third item; What is the trigger for considering the modification of the disease definition?

Our results should be interpreted with consideration of the study’s strengths and limitations. Our searches were limited by searching only one database with some reference checking. This search was not highly sensitive, and we may have missed some studies. However, we did find all of the studies that were reported in the literature review [14] and considered by the committee. Further, we assessed the quality of the available studies related to age of onset with relevant risk of bias tools and evaluated the body of research evidence related to the Checklist items.

While it is evident that the Committee endeavored to identify available research relating to the age of onset criterion by performing a literature review, details regarding study planning, the location and selection of available research, attempts to assess the quality of the research and to describe how it was taken into consideration was either not described or was not done. It is possible that methods were more rigorous than indicated in the publicly available document, but without adequate reporting we cannot be sure that the methods of the review were reliable and the conclusions trustworthy.

The available documentation indicates that the Committee considered the potential increase in prevalence of ADHD diagnosis as a result of the change to the age of onset criterion, but used the results of a single cohort twin study containing 68 children with ADHD [15] to conclude that the ‘impact on prevalence will be negligible’. Our assessment of this study found that the study design does not allow a full assessment of the impact of the change to the age of onset criterion on prevalence. While providing insight into potential changes in prevalence resulting from a change to the age of symptom onset, the effect on prevalence arising from both the change to age and impairment cannot be determined from this study. The prevalence change estimate from this study contrasts with a cross-sectional study in adolescents using retrospective recall of symptom onset by a parent, and a prospective cohort study of school aged children published after DSM-5, that report larger changes to prevalence with the new age of onset criterion [37, 38]. However, these studies also only evaluate the effect of the change in age threshold and not both the change in age and impairment requirements.

The available studies of prognosis and benefit or harm were generally at high risk of bias and did not assess the outcomes of, or potential benefits and harms of treatment, to those identified by the new but not old age of onset criterion. Further, no studies informed the precision of the new age of onset criterion. Again without studies considering the removal of the impairment requirement, as well as age of onset, the effect on prognostic ability from both changes cannot be fully elucidated or understood.

Changes to the way that health conditions are defined are likely to have widespread and significant consequences, and should be based on the highest quality of evidence possible. Proposed changes should be field tested in conditions that are as similar as possible to the conditions where the definition will be used, including community settings, to assess the effects on prevalence and diagnostic precision. Prognosis should be assessed by longitudinal cohort studies that have measured the elements used to define the criteria and have tracked clinical and other outcomes of interest over time. Benefits and harms from changes need to be carefully considered, preferably through the conduct of randomised controlled trials. It cannot be assumed that the benefits and harms seen in those diagnosed using a previous definition will apply to those diagnosed using the new definition.

Committees and panels need to report any research used to inform their decisions around changed definitions, critically assess and synthesise available research using systematic and justifiable methods, and provide this information to clinicians and relevant groups. The Checklist for Modifying Disease Definitions, a world-first evidence informed document, is designed to facilitate this process but is likely to require committee members with methodological and epidemiological expertise in its application.

## Conclusion

Changing the definition of health conditions places many people at risk of unnecessary diagnosis and treatment. This study found that changes to the DSM-IV ADHD age of onset criterion that widened the definition were based on research that was judged to be at high risk of bias and/or to have poor applicability. The research did not inform of the effects of changes to both the age and the impairment components of the criterion on the prevalence of ADHD. With such limited evidence on the effects of changes on prevalence, prognosis, precision, benefits and harms, the Committee could have chosen not to change the age of onset criterion, or, at the most, change the age component of the criterion, but retain the impairment component. Rigor and transparency in the processes around modifying health condition definitions should be expected. Committees and panels need to document reasons for any proposed changes, carefully report any research used to inform their decisions, and critically assess and synthesize this research using systematic and justifiable methods. As important as process, is the documentation of that process and details of decision making should be available to be critiqued by others. Although DSM-5 ADHD Committee members had provisions to include comments from the clinical and public communities through public postings, this document is no longer publicly available. When the documents were available, they were not detailed, and so decisions remain opaque. Revisions of DSM are regular, planned events. The Checklist should be used to inform further revisions and to facilitate the appropriate design and conduct of research that can address the effects of any future proposed changes.

## Supplementary information

**Additional file 1.** Contains additional tables and documents

## Data Availability

Data sharing is not applicable to this article as no datasets were generated or analysed during the study. A study protocol and the results of database searches and article screening can be obtained from the lead author on request. Additional files contain details of assessments of risk of bias and data extraction for each of the included studies.

## Abbreviation

ADHD: Attention deficit/hyperactivity disorder
AMSTAR-2: A measurement tool to assess systematic reviews
APA: American Psychiatric Association
DSM: Diagnostic and Statistical Manual of Mental Disorders
