# Supplementary material for: A review of changes to the attention deficit/hyperactivity disorder age of onset criterion using the checklist for modifying disease definitions
Source: BMC Psychiatry. 2019 Nov 12;19:357. doi: 10.1186/s12888-019-2337-7 (PMC6849294; doi:10.1186/s12888-019-2337-7)
Supplement: Supplementary file 1 — Additional file 1. Contains additional tables and documents [file 12888_2019_2337_MOESM1_ESM.docx]

**Additional file 1**

**Additional Tables**

**Table S1 Search strategy for identifying documents outlining the proposed or actual changes to the DSM-IV-TR ADHD age of onset criterion and any research used to inform the changes**

| PubMed search with Publication date from 1990/01/01 conducted on 21^st^ November 2017;  (ADHD[Title] OR attention deficit[Title] OR hyperactive*[Title] OR “Attention Deficit Disorder with Hyperactivity”[Mesh Terms])  AND  (DSM-IV[Title] OR DSM-5[Title] OR criteri*[Title] OR revis*[Title] OR (DSM-IV[Title/Abstract] AND DSM-5[Title/Abstract]))  Search terms used to search the American Psychiatric Association website on 21^st^ November 2017;  ‘DSM’  Search terms used to search Google Scholar on 21^st^ November 2017;  ADHD DSM “age of onset” Reviewed first 50 results  “Attention deficit hyperactivity” DSM “age of onset” Reviewed first 50 results  DSM-IV “age of onset” Reviewed first 50 results |
| --- |

**Table S2 PubMed search strategy for studies relating to the age of onset criterion that assists in answering the checklist items**

| PubMed search with Publication date from 1990/01/01 to 2013/01/01 conducted on 5^th^ January 2018;  (“Age of Onset”[MeSH Terms] OR “age of onset”[tiab] OR “age at onset”[tiab])  AND  (Attention Deficit Disorder with Hyperactivity[MeSH Terms] OR “attention deficit hyperactivity disorder”[tiab] OR “ADHD”[tiab] OR “ADDH”[tiab] OR “attention deficit disorder with hyperactivity”[tiab]) |
| --- |

**Table S3 Studies used by the Committee or identified in searches not assessed, with reason**

| **Studies used by the Committee not assessed (n=16)** | |
| --- | --- |
| **Study** | **Reason the study was not assessed** |
| Applegate 1997 | Not related to checklist items |
| Barkley 2006 | Not related to checklist items |
| Faraone 1998 | Not related to checklist items |
| Kuperman 2001 | Not related to checklist items |
| Lasky-Su 2007 | Not related to checklist items |
| Loeber 1992 | Not related to checklist items |
| Sullivan1990 | Not related to checklist items |
| Tillman 2003 | Not related to checklist items |
| Wisniewski 2007 | Not related to checklist items |
| Yang 2004 | Not related to checklist items |
| Barkley 1990 | Not in population of interest |
| Cuffe 2001 | Not in DSM version of interest |
| Kessler 2005 | Not estimating prevalence change resulting from change to age of onset criterion |
| Neuman 2005 | Not estimating prevalence change resulting from change to age of onset criterion |
| Barkley 2008 (2 studies in 1 publication) | Unable to locate the book in which these studies appear. Some study information could be located online but there was insufficient detail for assessment |
| **Studies identified in searches not assessed (n=7)** | |
| **Study** | **Reason the study was not assessed** |
| Applegate 1997 | Not related to checklist items |
| Neuman 2005 | Not related to checklist items |
| Kieling 2010 | Not a primary study |
| Samuel 1998 | Not a primary study |
| Barkley 1990 | Not in population of interest |
| Cuffe 2001 | Not in DSM version of interest |
| Miller 2010 | Not related to age of onset criterion changes specifically |

**Table S4 Assessment of risk of bias in the review (Kieling, 2010) presented in the Key Document**

| **AMSTAR 2 Criteria (Shea et al 2017)** | **Rating** | **Support for rating** |
| --- | --- | --- |
| 1. Did the research question and inclusion criteria include the components of PICO? | No | No research question was specified or reported |
| 1. Did the report of the review contain an explicit statement that the review methods were established prior to the conduct of the review and did the report justify any significant deviations from the protocol? | No | The existence of a protocol was not reported and could not be located |
| 1. Did the review authors explain their selection of study designs for inclusion in the review? | No | No explanation provided |
| 1. Did the review authors use a comprehensive literature search strategy? | Partial Yes | 2 databases were searched, search strings were provided. There were no publication restrictions. No other methods to locate studies were used or reported |
| 1. Did the review authors perform study selection in duplicate? | Yes |  |
| 1. Did the review authors perform study data extraction in duplicate? | No | Duplicate data extraction did not occur or was not reported |
| 1. Did the review authors provide a list of excluded studies and justify the exclusions? | No |  |
| 1. Did the review authors describe the Included studies in adequate detail? | No | Minimal detail of the studies was provided in supplementary table |
| 1. Did the review authors use a satisfactory technique for assessing the risk of bias (RoB) in individual studies that were included in the review? | No | Assessment of risk of bias did not occur or was not reported |
| 1. Did the review authors report on the sources of funding for the studies included in the review? | No |  |
| 1. If meta-analysis was performed did the review authors use appropriate methods for statistical combination of results? | No meta-analysis conducted |  |
| 1. If meta-analysis was performed, did the review authors assess the potential impact of RoB in individual studies on the results of the meta-analysis or other evidence synthesis? | No meta-analysis conducted |  |
| 1. Did the review authors account for RoB in individual studies when interpreting/discussing the results of the review? | No |  |
| 1. Did the review authors provide a satisfactory explanation for, and discussion of, any heterogeneity observed in the results of the review? | No | Exploration of heterogeneity did not occur or was not reported |
| 1. If they performed quantitative synthesis did the review authors carry out an adequate investigation of publication bias (small study bias) and discuss its likely impact on the results of the review? | No meta-analysis conducted |  |
| 1. Did the review authors report any potential sources of conflict of interest, including any funding they received for conducting the review? | No |  |

**Table S5a Prevalence studies: characteristics and results of studies assessing the effect of the change to the DSM-IV-TR age of onset criterion on the prevalence of ADHD**

| **Study** | **Design features** | **Result** | **Comment** |
| --- | --- | --- | --- |
|  | | |  |
| Polanczyk,  2010 | Design: Prospective cohort  Participants: 2232 British twins  ADHD diagnosis comprises: ≥6 inattentive and/or ≥ 6 hyperactivity-impulsivity DSM-IV symptoms (in preceding 6 months) on mother interview or teacher questionnaire + endorsement of ≥ 2 symptoms meeting pervasiveness criterion by other informant | At age 12, 66 children met study ADHD criteria with onset of symptoms before age 7. An additional 2 children met study ADHD criteria with onset of symptoms between 7 and 12 years of age. Prevalence increase 0.1%. | Effect of change to both the age and impairment requirement was not considered  Twins were the population studied  Prevalence of ADHD was low  Symptom onset was prospectively measured.  No estimate of the uncertainty around the prevalence increase was provided |

**Table S5b Prevalence studies: risk of bias in studies assessing the effect of the change to the DSM-IV TR age of onset criterion on the prevalence of ADHD**

| **Study** | **Risk of Bias Domains for studies of prevalence (Hoy et al, 2012)** | | | | | | | | | | |
| --- | --- | --- | --- | --- | --- | --- | --- | --- | --- | --- | --- |
|  | External validity | | | | Internal validity | | | | | | |
|  | 1. Was the study’s target population a close representation of the national population in relation to relevant variables? | 2. Was the sampling frame a true or close representation of the target population? | 3. Was some form of random selection used to select the sample, OR, was a census undertaken? | 4. Was the likelihood of non-response bias minimal? | 5.Were data collected directly from subjects? | 6. Was an acceptable case definition used in the study? | 7. Was the study instrument that measured the parameter of interest shown to have reliability and validity (if necessary)? | 8. Was the same mode of data collection used for all subjects? | 9. Was the length of the shortest prevalence period for the parameter of interest appropriate? | 10. Were the numerator(s) and denominator(s) for the parameter of interest appropriate? |  |
| Polanczyk ,  2010 | No (High risk) | Yes (Low risk) | Yes (Low risk) | Yes (Low risk) | Yes (Low risk) | Yes (Low risk) | Yes (Low risk) | Yes (Low risk) | Yes (Low risk) | Yes (Low risk) |  |

**Table S6a Prognosis studies: characteristics and results of studies assessing the prognostic ability of the DSM-5 age of onset criterion**

| **Study** | **Design features** | **Result*** | **Comment** |
| --- | --- | --- | --- |
| Cohort studies | | | |
| McGee 1992 | P: children in a birth cohort study  C: symptoms present (by DSM-III criteria) at age 3, age 5-6, and age 7  O: behavior problems, comorbidity, cognitive and reading ability, presence of disorder at later age | “Onset was strongly related to informant source at age 11, pattern of comorbidity of disorder at age 11, and developmental language, perceptual motor, and IQ measures.” | Prognosis in additional patients unknown^#^ |
| Willoughby 2000 | P: children in a community based survey  C: a) early onset of ADHD symptoms; b) late onset of ADHD symptoms, by subtypes  O: impairment, service use, comorbidity | “Early onset of ADHD symptoms was associated with worse clinical outcomes in youths with the combined subtype of ADHD but not youths with the inattentive subtype.” | Prognosis in additional patients unknown^#^ |
| Cross sectional studies | | | |
| Connor 2003 | P: children newly diagnosed with ADHD attending an ADHD clinic  C: age of onset of impairing symptoms (by DSM-IV criteria) at 0-2 years, 3-4 years, 5-6 years and >6 years  O: comorbid symptoms | “…our results suggest that the presence of comorbid symptoms is influenced by age of onset. An early age of onset of ADHD was correlated with a greater rate of parent-reported child aggressive symptoms, and a later age of onset was correlated with a greater rate of parent-reported child anxious/depressive symptoms.” | Prognosis in additional patients unknown^#^ |
| Faraone 2006(a), 2006(b), 2007, 2009 | P: adults referred to psychiatric clinics or responding to advertisements  C: a) met all DSM-IV ADHD criteria (‘full ADHD’); b) met DSM-IV ADHD criteria except age at onset criterion (‘late onset’); c) subthreshold ADHD; d) no ADHD  O: comorbidity, functional impairment, familial transmission (2006(a)); neuropsychological functioning (2006 (b)); substance use (2007); personality profile (2009) | ‘late onset and full ADHD had similar patterns of psychiatric comorbidity, functional impairment, and familial transmission.’  ‘Late-onset and full ADHD subjects had similar patterns of neuropsychological dysfunction.”  No difference in one month prevalence of any use of alcohol, marijuana or illicit drugs between late onset and full ADHD group. One month prevalence of any use of cigarettes was significantly greater in the late-onset ADHD group than the full ADHD group.  “We found that full ADHD and late-onset ADHD showed similar personalities profiles.” | Prognosis in additional patients unknown^#^,  ‘late’ onset group had a range of onset of 7-45 years. |
| Guimaraes-da-Silva 2012 | P: adults attending an ADHD outpatient clinic  C: a) met DSM-IV criteria for ADHD (‘early onset’); b) met DSM-IV criteria except age of onset criterion (‘late onset’)  O: neuropsychological and personality characteristics | “Patients with early onset ADHD present higher scores in novelty seeking in both analyses (respectively P=0.016 and P=0.002), but similar cognitive and attention features as compared with the late onset group.” | Prognosis in additional patients unknown^#^,  Possibly includes participants from the same study population as presented in Karam, 2009. |
| Hesslinger 2003 | P: adults attending an outpatient ADHD clinic  C: a) met DSM-IV criteria for ADHD between age 6 and 10 years (‘early onset ADHD’); b) Did not fulfill criteria for ADHD between 6 and 10 years, but did in later age ranges 11 to 18 years (‘late onset ADHD’)  O: psychopathology, psychiatric comorbidity | “There was no difference between early onset and late onset ADHD groups in terms of psychopathology or psychiatric comorbidity.” | Prognosis in additional patients unknown^#^ |
| Karam 2009 | P: adults attending an outpatient ADHD clinic  C: a) met DSM-IV criteria for ADHD including age of onset of impairing symptoms before 7 years (‘early onset’); b) met all DSM-IV criteria for ADHD except onset of impairing symptoms occurred between 7 and 12 years (‘late onset’).  O: age at diagnosis, social-economic status, severity of symptoms, comorbidity profile | “Late onset subjects were diagnosed later (p=0.04), had a lower frequency of problems with authority and discipline (p=0.004), and lower scores in SNAP-IV (p<0.001) and in Barkley’s scale for problems in areas of life activities (p=0.03). On the other hand, late-onset patients presented a higher prevalence of comorbid general anxiety disorder (GAD) (P=0.01). Both groups had a similar profile in the remaining comorbidities and sociodemographic characteristics.” | Prognosis in additional patients unknown^#^ |
| Ruckledge 2002 | P: subset of adolescents from a study of ADHD gender differences  C: a) ADHD symptoms present but not past (‘adolescent-onset ADHD’); b) childhood-onset persisting ADHD; c) ADHD past but not present; d) no ADHD past or present  O: cognitive performance | “…adolescents with childhood-onset ADHD were slower in processing speed (Symbol Search), color naming, and number naming and were more variable in response times and accuracy of responses (as measured by the Stop-Signal test) compared with the ‘adolescent-onset ADHD’ group.” | Prognosis in additional patients unknown^#^ |
| Rohde 2000 | P: adolescents at school  C: a) met all DSM-IV ADHD criteria; b) met all DSM-IV ADHD criteria except age of onset criterion; c) no ADHD  O: symptoms, global impairment | “Adolescents with ADHD and youths with ADHD w/o age-of-onset did not differ significantly in any measure assessed.” | Prognosis in additional patients unknown^#^ |
| Waschbusch 2007 | P: school students  C: a) met DSM-IV ADHD criteria including age of onset criterion; b) fulfilled DSM-IV ADHD criteria but did not meet age of onset criterion  O: Impairment | “Groups did not differ significantly on number of inattention symptoms… number of hyperactive/impulsive symptoms… impairment from inattention…, or impairment from hyperactivity/impulsivity. ADHD children who did not meet the age on onset criterion were rated as having significantly more impaired parent-child relationships, self-esteem, family functioning and as having significantly more overall impairment as compared to ADHD children who met the age of onset criterion.” | Prognosis in additional patients unknown^#^ |
| Willcutt 2012 | P: school children  C: a) children with ADHD according to DSM-IV with onset of symptoms before age 7; b) children with ADHD according to DSM-IV except with onset of symptoms after age 7  O: functional impairment | “…the rate of functional impairment was almost identical in the groups of children with ADHD with onset before and after age 7 (e.g., 80% vs 82% of cases in the 2 groups exhibited cross-setting impairment when ADHD symptoms were defined by best estimate procedures).” | Prognosis in additional patients unknown^#^ |

* results presented are a summary of study findings as stated by the study investigators; P population; C comparison; O outcome; # the study does not compare outcomes in those labelled by the new but not old age of onset criterion (the ‘additional’ patients identified by the new criterion), and the old age of onset criterion

**Table S6b** **Prognosis studies:** **risk of bias in studies assessing the prognostic ability of the DSM-5 age of onset criterion**

| **Study** | **Risk of Bias Domains according to Quality in Prognosis Study (QUIPS) tool (Hayden et al 2013)** | | | | | |
| --- | --- | --- | --- | --- | --- | --- |
|  | Study participation | Study attrition | Prognostic factor measurement | Outcome measurement | Study confounding | Statistical analysis and reporting |
| Cohort studies | | | | | | |
| McGee 1992 | Low | Moderate | Low | Moderate | High | Moderate |
| Willoughby 2000 | Low | Moderate | Low | Low | Moderate | Moderate |
| Cross sectional studies | | | | | | |
| Connor 2003 | Moderate | Moderate | Moderate | Low | Low | Moderate |
| Farone 2006(a), 2006(b), 2007, 2009 | Moderate | Moderate | Moderate | Low | Moderate | Moderate |
| Guimaraes-da-Silva 2012 | Moderate | Moderate | Moderate | Low | Low | Moderate |
| Hesslinger 2003 | Low | Low | Moderate | Moderate | High | Moderate |
| Karam 2009 | Moderate | Moderate | Moderate | Low | Low | Moderate |
| Rohde 2000 | Low | Low | Moderate | Low | High | Moderate |
| Ruckledge 2002 | High | High | Moderate | Moderate | Low | Moderate |
| Waschbush 2007 | Moderate | High | Moderate | Low | Moderate | Moderate |
| Wilcutt 2012 | Moderate | Low | Moderate | Low | High | Moderate |

**Table S7a Precision studies: characteristics and results of studies assessing the precision of the DSM-5 age of onset criterion**

| **Study** | **Design features** | **Results** | **Comments** |
| --- | --- | --- | --- |
|  | | | |
| Angold 1996 | 8-18 year old’s reported date of onset of individual symptoms at interviews with different interviewers 1 week apart | “When symptoms have lasted longer than around 3 months, the month of onset usually cannot be accurately reported. The ICC for 380 symptoms reported on both occasions was 0.49. When symptoms present for longer than 3 months, around only 20% of onset pair dates were within 30 days of each other | Study does not address checklist item* |
| Green 1991 | Mothers of children aged 7-12 questioned about age of onset of DSM-III behaviours using the Diagnostic Interview Schedule for Children (DISC) at baseline and 1 year later | “The data shows a moderate degree of stability over a 1-year period”. The mean age of onset for year 1 was not significantly different from the mean age of onset for Year 2. Median correlation for age of onset for all symptoms was 0.62. The median percentage of subjects recalling the same age of onset between first and second assessment was 33.3%. | Study does not address checklist item* |
| Todd 2008 | Twins aged 7-19 years and parents meeting a symptom screening interview reporting age of onset of individual symptom impairment at baseline and 5 years later | ‘Later ages of ADHD onset were reported 5 years later’  Mean age of onset by parent report at time 1 was 3.6 years and at time 2, 4.2 years. Mean age of onset by self-report at time 1 was 4.6 years and at time 2, 5.6 years. | Study does not address checklist item* |

* the study does not assess the repeatability (agreement between the same clinicians at different times) or reproducibility (agreement between different clinicians) of clinicians judgments that an individual does or does not meet the new age of onset criterion

**Table S7b Precision studies: risk of bias in studies assessing the precision of the DSM-5 age of onset criterion**

| Study | **Risk of Bias Domain according to the QAREL tool for studies of diagnostic reliability (Lucas 2009)** | | | | | | | | | | |
| --- | --- | --- | --- | --- | --- | --- | --- | --- | --- | --- | --- |
|  | Was the test evaluated in a sample of subjects representative of those considered ideal for evaluating the reliability of the age of onset criteria* | Was the test performed by raters# who were representative of those to whom the authors intended the results to be applied? | Were raters# blinded to the findings of other raters during the study? | Were raters# blinded to their own prior findings of the test under evaluation?~ | Were raters# blinded to the results of the reference standard for the target disorder (or variable) being evaluated? | Were raters #blinded to clinical information that was not intended to be provided as part of the testing procedure or study design? | Were raters# blinded to additional cues that were not part of the test? | Was rater^ the order of examination varied? | Was the time interval between repeated measurements compatible with the stability (or theoretical stability) of the variable being measured? | Was the test applied correctly and interpreted appropriately? | Were appropriate statistical measures of agreement used? |
| Green 1991 | Unclear | Unclear | Unclear | NA | NA | No | No | NA | Yes | Yes | Yes |
| Angold 1996 | Unclear | No | Yes | NA | NA | No | No | NA | Yes | Yes | Yes |
| Todd 2008 | No | Unclear | Yes | NA | No | No | No | NA | Yes | Yes | No |

*this item was modified from the original checklist. We considered a representative sample to be study participants who were attending a clinic for initial evaluation of suspected ADHD rather than populations with diagnosed ADHD

#raters are the individuals conducting the interviews at which age of onset is obtained from study participants

~this item was not applicable if raters only examined a subject once.

^the word rater was added to this checklist item to clarify it related to the order of raters doing the examination rather than the examination itself i.e. in what order information was collected during an interview.

**Table S8a Benefits and Harms studies: characteristics and results of studies assessing benefits and/or harms**

| **Study** | **Design features** | **Results** | **Comments** |
| --- | --- | --- | --- |
| Biederman 2006 | P: 36 adults with ADHD not otherwise specified (by DSM-IV criteria) attending outpatient ADHD clinic  I/: Methylphenidate up to 1.3mg/kg/day; no comparator  O: symptom severity assessed with Adult ADHD Investigator Symptom Report Scale and the Clinical Global Impression Scale at 6 weeks after start of treatment | Significant reduction in symptoms of inattention and hyperactivity from baseline to week 6  72% of participants were ‘much’ or ‘very much’ improved at 6 weeks  Adverse effects were commonly reported | Not incremental |
| Reinhardt 2007 | P: 180 children, adolescents and 111 adults attending a hospital clinic meeting DSM-IV ADHD criteria except age of onset criterion  I/C: Methylphenidate minimum dose 0.30mg/kg/day; no comparator  O: response to treatment (according to SNAP-IV scale) in those meeting the age of onset criterion and those not meting age on onset criterion at 1 month after start of treatment | In children and adolescents there was no difference in response to methylphenidate between those with full and late-onset ADHD  Adults with late onset had a significantly higher response to methylphenidate than those with full ADHD (after adjustment for confounders p=0.018) | Not incremental |

*the study does not assess treatment benefits and harms in individuals diagnosed by the new but not old age of onset criterion; P population; I intervention; C comparator; O outcome

**Table S8b Benefits and Harms studies: risk of bias in studies assessing benefits and/or harms**

|  | **Risk of Bias Domains according to a tool for assessing risk of bias in single-arm studies included in systematic reviews (Beller, E. Personal communication)** | | | | | | | | | | | |
| --- | --- | --- | --- | --- | --- | --- | --- | --- | --- | --- | --- | --- |
| Study | Selection bias | | | Lead time bias/  immortal time bias | | Confounding by indication | Misclassification bias/ information bias | | Bias from natural recovery/ regression to the mean | Bias due to adjunct therapies | Attrition bias* | Selective reporting of outcomes* |
|  | 1. Was the selection of participants either consecutive or randomly selected from the population? | 2A. Were the eligibility criteria clearly described? | 2B. If yes, were the eligibility criteria similar to the other studies in the review that had a control group | 3.Was the time between starting follow-up for outcomes (recruitment) and starting the intervention of an appropriately short duration? | 4.Was it similar to other studies in the review that had a control group? | 5. Are those in the study at a similar stage/severity of their disease and have similar prognostic factors to other studies in the review that had a control group? | 6.Was dose (or other details) of intervention, both planned and given, clearly described? | 7. Was measurement of outcome made by a reliable and valid method (e.g. objective measure)? | 8. Were outcome variables measured pre-intervention (i.e. interrupted time series design with multiple measurements, of before-after design)? | 9. Is there adequate reporting of adjunctive therapies both before and during the study protocol? | Incomplete outcome data | Selective outcome reporting |
| Biederman  2006 | Can’t tell/not reported | Yes | NA | Can’t tell/not reported | NA | NA | Yes | Yes | Yes | Yes | High | Unclear |
| Reinhardt  2007 | Can’t tell/not reported | Yes | NA | Can’t tell/not reported | NA | NA | No | Yes | Yes | Can’t tell/not reported | High | Unclear |

*item is from The Cochrane Collaboration’s tool for assessing risk of bias in randomised trials where judgements about the risk of bias are rated as ‘High risk’, ‘Unclear risk’, or ‘Low risk’.

NA not applicable

References to studies presented in additional tables

Angold A, Erkanli A, Costello EJ, Rutter M. Precision, reliability and accuracy in the dating of symptom onsets in child and adolescent psychopathology. J Child Psychol Psychiatry. 1996;37(6):657-64.

Applegate B, Lahey BB, Hart EL, Biederman J, Hynd GW, Barkley RA, et al. Validity of the age-of-onset criterion for ADHD: a report from the DSM-IV field trials. J Am Acad Child Adolesc Psychiatry. 1997;36(9):1211-21.

Barkley RA, Fischer M, Edelbrock CS, Smallish L. The adolescent outcome of hyperactive children diagnosed by research criteria: I. An 8-year prospective follow-up study. J Am Acad Child Adolesc Psychiatry. 1990;29(4):546-57.

Barkley RA, Murphy KR, Fischer M. ADHD in Adults: What the science says. New York: Guilford Press; 2008.

Barkley RA, Smith KM, Fischer M, Navia B. An examination of the behavioral and neuropsychological correlates of three ADHD candidate gene polymorphisms (DRD4 7+, DBH TaqI A2, and DAT1 40 bp VNTR) in hyperactive and normal children followed to adulthood. Am J Med Genet B Neuropsychiatr Genet. 2006;141B(5):487-98.

Biederman J, Mick E, Spencer T, Surman C, Hammerness P, Doyle R, et al. An open-label trial of OROS methylphenidate in adults with late-onset ADHD. CNS Spectr. 2006;11(5):390-6.

Connor DF, Edwards G, Fletcher KE, Baird J, Barkley RA, Steingard RJ. Correlates of comorbid psychopathology in children with ADHD. J Am Acad Child Adolesc Psychiatry. 2003;42(2):193-200.

Cuffe SP, McKeown RE, Jackson KL, Addy CL, Abramson R, Garrison CZ. Prevalence of attention-deficit/hyperactivity disorder in a community sample of older adolescents. J Am Acad Child Adolesc Psychiatry. 2001;40(9):1037-44.

Faraone SV, Biederman J, Doyle A, Murray K, Petty C, Adamson JJ, et al. Neuropsychological studies of late onset and subthreshold diagnoses of adult attention-deficit/hyperactivity disorder. Biol Psychiatry. 2006;60(10):1081-7.

Faraone SV, Biederman J, Spencer T, Mick E, Murray K, Petty C, et al. Diagnosing adult attention deficit hyperactivity disorder: are late onset and subthreshold diagnoses valid? Am J Psychiatry. 2006;163(10):1720-9; quiz 859.

Faraone SV, Biederman J, Weber W, Russell RL. Psychiatric, neuropsychological, and psychosocial features of DSM-IV subtypes of attention-deficit/hyperactivity disorder: results from a clinically referred sample. J Am Acad Child Adolesc Psychiatry. 1998;37(2):185-93.

Faraone SV, Kunwar A, Adamson J, Biederman J. Personality traits among ADHD adults: implications of late-onset and subthreshold diagnoses. Psychol Med. 2009;39(4):685-93.

Faraone SV, Wilens TE, Petty C, Antshel K, Spencer T, Biederman J. Substance use among ADHD adults: implications of late onset and subthreshold diagnoses. Am J Addict. 2007;16 Suppl 1:24-32; quiz 3-4.

Green SM, Loeber R, Lahey BB. Stability of mothers' recall of the age of onset of their child's attention and hyperactivity problems. J Am Acad Child Adolesc Psychiatry. 1991;30(1):135-7.

Guimaraes-da-Silva PO, Silva KL, Grevet EH, Salgado CA, Karam RG, Victor MM, et al. Does age of onset of impairment impact on neuropsychological and personality features of adult ADHD? J Psychiatr Res. 2012;46(10):1307-11.

Hayden JA, van der Windt DA, Cartwright JL, Cote P, Bombardier C. Assessing bias in studies of prognostic factors. Annals of internal medicine. 2013;158(4):280-6.

Hesslinger B, Tebartz van Elst L, Mochan F, Ebert D. Attention deficit hyperactivity disorder in adults-early vs. late onset in a retrospective study. Psychiatry Res. 2003;119(3):217-23.

Hoy D, Brooks P, Woolf A, Blyth F, March L, Bain C, et al. Assessing risk of bias in prevalence studies: modification of an existing tool and evidence of interrater agreement. Journal of clinical epidemiology. 2012;65(9):934-9.

Karam RG, Bau CH, Salgado CA, Kalil KL, Victor MM, Sousa NO, et al. Late-onset ADHD in adults: milder, but still dysfunctional. J Psychiatr Res. 2009;43(7):697-701.

Kessler RC, Berglund P, Demler O, Jin R, Merikangas KR, Walters EE. Lifetime prevalence and age-of-onset distributions of DSM-IV disorders in the National Comorbidity Survey Replication. Arch Gen Psychiatry. 2005;62(6):593-602.

Kieling C, Kieling RR, Rohde LA, Frick PJ, Moffitt T, Nigg JT, et al. The age at onset of attention deficit hyperactivity disorder. Am J Psychiatry. 2010;167(1):14-6.

Kuperman S, Schlosser SS, Kramer JR, Bucholz K, Hesselbrock V, Reich T, et al. Developmental sequence from disruptive behavior diagnosis to adolescent alcohol dependence. Am J Psychiatry. 2001;158(12):2022-6.

Lasky-Su J, Biederman J, Laird N, Tsuang M, Doyle AE, Smoller JW, et al. Evidence for an association of the dopamine D5 receptor gene on age at onset of attention deficit hyperactivity disorder. Ann Hum Genet. 2007;71(Pt 5):648-59.

Loeber R, Green SM, Lahey BB. Developmental sequences in the age of onset of disruptive child behaviors. J Child Fam Stud. 1992;1:21-41.

Lucas NP, Macaskill P, Irwig L, Bogduk N. The development of a quality appraisal tool for studies of diagnostic reliability (QAREL). Journal of clinical epidemiology. 2010;63(8):854-61.

McGee R, Williams S, Feehan M. Attention deficit disorder and age of onset of problem behaviors. J Abnorm Child Psychol. 1992;20(5):487-502.

Miller CJ, Newcorn JH, Halperin JM. Fading memories: retrospective recall inaccuracies in ADHD. J Atten Disord. 2010;14(1):7-14.

Neuman RJ, Sitdhiraksa N, Reich W, Ji TH, Joyner CA, Sun LW, et al. Estimation of prevalence of DSM-IV and latent class-defined ADHD subtypes in a population-based sample of child and adolescent twins. Twin Res Hum Genet. 2005;8(4):392-401.

Polanczyk G, Caspi A, Houts R, Kollins SH, Rohde LA, Moffitt TE. Implications of extending the ADHD age-of-onset criterion to age 12: results from a prospectively studied birth cohort. J Am Acad Child Adolesc Psychiatry. 2010;49(3):210-6.

Reinhardt MC, Benetti L, Victor MM, Grevet EH, Belmonte-de-Abreu P, Faraone SV, et al. Is age-at-onset criterion relevant for the response to methylphenidate in attention-deficit/hyperactivity disorder? J Clin Psychiatry. 2007;68(7):1109-16.

Rohde LA, Biederman J, Zimmermann H, Schmitz M, Martins S, Tramontina S. Exploring ADHD age-of-onset criterion in Brazilian adolescents. Eur Child Adolesc Psychiatry. 2000;9(3):212-8.

Rucklidge JJ, Tannock R. Age of onset of ADHD symptoms. J Am Acad Child Adolesc Psychiatry. 2002;41(5):496-7.

Sameul RZ. Age of onset for ADHD. J Am Acad Child Adolesc Psychiatry. 1998;37(6):569-70.

Shea BJ, Reeves BC, Wells G, Thuku M, Hamel C, Moran J, et al. AMSTAR 2: a critical appraisal tool for systematic reviews that include randomised or non-randomised studies of healthcare interventions, or both. Bmj. 2017;358:j4008.

Sullivan A, Kelso J, Stewart M. Mothers' views on the ages of onset for four childhood disorders. Child Psychiatry Hum Dev. 1990;20(4):269-78.

Tillman R, Geller B, Bolhofner K, Craney JL, Williams M, Zimerman B. Ages of onset and rates of syndromal and subsyndromal comorbid DSM-IV diagnoses in a prepubertal and early adolescent bipolar disorder phenotype. J Am Acad Child Adolesc Psychiatry. 2003;42(12):1486-93.

Todd RD, Huang H, Henderson CA. Poor utility of the age of onset criterion for DSM-IV attention deficit/hyperactivity disorder: recommendations for DSM-V and ICD-11. J Child Psychol Psychiatry. 2008;49(9):942-9.

Waschbusch D, King S, Gregus A. Age of onset of ADHD in a sample of elementary school students. J Psychopathol Behav Assess. 2007;29:9-16.

Willcutt EG. The prevalence of DSM-IV attention-deficit/hyperactivity disorder: a meta-analytic review. Neurotherapeutics. 2012;9(3):490-9.

Willoughby MT, Curran PJ, Costello EJ, Angold A. Implications of early versus late onset of attention-deficit/hyperactivity disorder symptoms. J Am Acad Child Adolesc Psychiatry. 2000;39(12):1512-9.

Wisniewski KG. Delinquency, academic underachievement, and attention deficit hyperactivity disorder: a longitudinal investigation of developmental sequencing an dinterrelated risk factors: Duquesne University; 2007.

Yang L, Wang YF, Qian QJ, Biederman J, Faraone SV. DSM-IV subtypes of ADHD in a Chinese outpatient sample. J Am Acad Child Adolesc Psychiatry. 2004;43(3):248-50.

**Additional Document**

(Accessed from http://www.dsm5.org/ProposedRevisions/Pages/proposedrevision.aspx?rid=383# 2012)

# A 06 Attention Deficit/Hyperactivity Disorder

- Proposed  Revision
- Rationale
- Severity
- DSM-IV

Updated May 1, 2012



AD/HD consists of a pattern of behavior that is present in multiple settings where it gives rise to social, educational or work performance difficulties.

**A.** Either (A1) and/or (A2).

**A1. Inattention**: Six (or more) of the following symptoms have persisted for at least 6 months to a degree that is inconsistent with developmental level and that impact directly on social and academic/occupational activities.

a.   Often fails to give close attention to details or makes careless mistakes in schoolwork, at work, or during other activities (e.g., overlooks or misses details, work is inaccurate).

b.   Often has difficulty sustaining attention in tasks or play activities (e.g., has difficulty remaining focused during lectures, conversations, or reading lengthy writings).

c.   Often does not seem to listen when spoken to directly (e.g., mind seems elsewhere, even in the absence of any obvious distraction).

d.   Often does not follow through on instructions and fails to finish schoolwork, chores, or duties in the workplace (e.g., starts tasks but quickly loses focus and is easily sidetracked;  fails to finish schoolwork, household chores, or tasks in the workplace).

e.   Often has difficulty organizing tasks and activities (e.g., difficulty managing sequential tasks; difficulty keeping materials and belongings in order; messy, disorganized, work; poor time management; tends to fail to meet deadlines).

f.    Often avoids, dislikes, or is reluctant to engage in tasks that require sustained mental effort (e.g., schoolwork or homework; for older adolescents and adults, preparing reports, completing forms, or reviewing lengthy papers).

g.   Often loses things necessary for tasks or activities (e.g., school materials, pencils, books, tools, wallets, keys, paperwork, eyeglasses, or mobile telephones).

h.   Is often easily distracted by extraneous stimuli (for older adolescents and adults, may include unrelated thoughts).

i.    Is often forgetful in daily activities (e.g., chores, running errands; for older adolescents and adults, returning calls, paying bills, keeping appointments).

**A2. Hyperactivity and Impulsivity:** Six (or more) of the following symptoms have persisted for at least 6 months to a degree that is inconsistent with developmental level and that impact directly on social and academic/occupational activities.

a.   Often fidgets with or taps hands or feet or squirms in seat.

b.   Often leaves seat in situations when remaining seated is expected (e.g., leaves his or her place in the classroom, office or other workplace, or in other situations that require remaining seated).

c.   Often runs about or climbs in situations where it is inappropriate. (In adolescents or adults, may be limited to feeling restless).

d.   Often unable to play or engage in leisure activities quietly.

e.   Is often “on the go,” acting as if “driven by a motor” (e.g., is unable or uncomfortable being still for an extended time, as in restaurants, meetings, etc; may be experienced by others as being restless and difficult to keep up with).

f.    Often talks excessively.

g.   Often blurts out an answer before a question has been completed (e.g., completes people’s sentences and “jumps the gun” in conversations, cannot wait for next turn in conversation).

h.   Often has difficulty waiting his or her turn (e.g., while waiting in line).

i.    Often interrupts or intrudes on others (e.g., butts into conversations, games, or activities; may start using other people’s things without asking or receiving permission, adolescents or adults may intrude into or take over what others are doing).

B.   Several inattentive or hyperactive-impulsive symptoms were present prior to age 12.

C.   Criteria for the disorder are met in two or more settings (e.g., at home, school or work, with friends or relatives, or in other activities).

D.   There must be clear evidence that the symptoms interfere with or reduce the quality of social, academic, or occupational functioning.

E.   The symptoms do not occur exclusively during the course of schizophrenia or another psychotic disorder and are not better accounted for by another mental disorder (e.g., mood disorder, anxiety disorder, dissociative disorder, or a personality disorder).

Specify Based on Current Presentation

**Combined Presentation:** If both Criterion A1 (Inattention) and Criterion A2 (Hyperactivity-Impulsivity) are met for the past 6 months.

**Predominantly Inattentive Presentation Rationale for Changes in ADHD in DSM-5**

**From the ADHD and Disruptive Behavior Disorders Workgroup**

Several potential problematic issues with DSM-IV ADHD criteria were not addressed due to lack of evidence based data and available resources/time to produce it. So, these issues  are deferred to future revisions of the DSM. However, the work group felt comfortable with the level of evidence available to tackle some issues. The following changes are proposed for ADHD in DSM-5.

1) Change the age of onset from onset of impairing symptoms by age 7 to onset of symptoms by age 12,

2) Change the three subtypes to three current presentations;

3) Add a fourth presentation for restrictive inattentive;

4) Change the examples in the items, without changing the exact wording of the DSM-IV items, to accommodate a lifespan relevance of each symptom and to improve clarity.

6) Remove PDD from the exclusion criteria.

7) Modify the pre-amble A1 and A2 to indicate that information must be obtained from two different informants (parents and teachers for children and third part/significant other for adults) whenever possible.

8) Still under consideration: Adjust the cut point for diagnosis in adults.

**1) Change Age of Onset**. The detailed justification for this change is found in the appended report which summarizes the results of a systematic literature review conducted by the workgroup and published by the workgroup age-of-onset subcommittee (Kieling et al., 2010); appendix A). Further evidence was summarized in Polanczyk et al. (Polanczyk et al., 2010). The reasoning and data are summarized here.

**a. Magnitude of Change**. Moderate change.

**b. Reason/Evidence for Change.** A substantial literature since 1994 overwhelmingly indicated that the age of onset by age 7 was invalid by all criteria: not able to be reliably assessed, no clinical differences between children identified as onset by 7 versus later in terms of course, severity, outcome, or treatment response (Kieling, et al., 2010); Appendix A-1). Whereas age 7 has no validity, some limits to age of onset are needed to maintain the conception of ADHD as a developmental condition and the conception that new onset in adolescence or adulthood has not been validated as ADHD. The DSM-IV field trial data published subsequently to DSM-IV showed that valid cases of ADHD did not always meet the age of onset at 7 (Applegate et al., 1997). Other data in the literature indicate that 96% of lifetime cases of ADHD are captured/onset by age 12-14 (R. A. Barkley & Brown, 2008), suggesting that an age 12 cutoff is superior to most alternatives. With renewed concern about appropriate criteria for adults, issues of retrospective recall also were clinically relevant. Population survey data indicated that in adults with ADHD, only half recalled onset by age 7 but 95% recalled onset by age 12 (R. A. Barkley & Brown, 2008).

**c. Potential negative consequences considered**. The main potential negative consequence of raising the age of onset is an increase in prevalence. The work group considered this possibility with some concern. However, data analysis on a British birth cohort published by the age-of-onset subcommittee suggest that the impact on prevalence will be negligible  (Polanczyk, et al., 2010).

**d. Additional objections and response.** Another objection raised by some authorities was that the age of onset of 12 would remain arbitrary and could provide a false sense of precision; this viewpoint proposed that the age of onset should in fact be eliminated entirely or should be raised to age 18 (or “onset in childhood). However, as noted, the data we were able to obtain indicated that 96% of cases are captured by age 12-14, suggesting that an age 12 cutoff is superior to most alternatives. Furthermore, the workgroup members concluded that from a conceptual and clinical point of view, new onset in mid adolescence was not validated as consistent with ADHD for the great majority of cases. A general specifier of “onset in childhood” was considered by the workgroup, but was deemed too vague and as potentially creating an impediment to research sample collection while failing to add benefit to clinical practice versus the age 12 onset. The text explains that just like age 7, age 12 is a clinical guideline meant to convey the developmental onset.

Second, there is ongoing debate whether to require impairment by the age-of onset (as in DSM-IV) or symptom onset by age-of-onset (as now proposed for DSM-5). There is a general notion in the field that the latter are more likely to depend on environmental demands, such as school. The newly worded onset criterion explicitly gives the message that a clear set of symptoms should be presented in childhood. This is consonant with the notion of ADHD as a neurodevelopmental disorder. Unfortunately, there is no evidence to guide the decision on the exact number of symptoms that should be required in the “by-age-of-onset” period (for a full discussion on this issue see: (Matte, Grevet, & Rohde, in press). This proposal is also consonant with the conceptual decision to differentiate assessments of symptoms and impairment in the DSM-5. It also renders ADHD diagnosis more compatible between DSM and ICD systems since ICD requires age-of-onset and not age-of-onset of impairment.

**2) Change subtypes to a specifier for presentations, and (3) add a fourth presentation for restrictive inattentive.** This change has two parts: changing from subtype to presentation, and adding a fourth option. The detailed literature review supporting this change is included in Appendix B (Willcutt, Nigg, Rohde, Tannock, & Solanto, in press). Key findings are summarized here.

**a. Magnitude of Change: Moderate Change**.

**b. Reason for Change.** Neuropsychological, comorbidity, treatment response, and clinical outcome data suggest that there is some variation between DSM-IV ADHD subtypes related to their variation on the underlying symptom dimensions, but this is a variation in degree, not in kind. In other words, “subtypes” are essentially functioning as shorthand for conveying dimensional variation in risk at the current time (based on variation in inattention versus in hyperactivity-impulsivity). However, they do not convey distinct characteristics. Longitudinal studies in four samples, albeit each with important methodological limitations, concur that the putative types are not stable, but change into one another in non-lawful manner (Lahey, Pelham, Loney, Lee, & Willcutt, 2005); reviewed in (Willcutt, et al., in press). Thus, it was concluded that subtypes denotes a stable difference, whereas presentation denotes a current presentation that will be less reifying than subtypes.

However, eliminating a concept of heterogeneity entirely was, after much discussion, judged as premature because (a) brain imaging and genetic data are still lacking on the presentations or subtypes and thus could overturn the idea that subtypes are not biologically distinct (Wilcutt et al), (b) family history data still suggest partial though weak familial transmission and it remains unclear if this is explained by transmission of symptom domain (Stawicki, Nigg, & von Eye, 2006), and (c) it would be damaging to DSM credibility to fully eliminate these characterizations in DSM-5 and potentially be forced to reinstate them in DSM6 when more biological data are available.

Furthermore, there was a perceived lack of literature on a clinically and theoretically important group of children with high levels of inattention but essentially no hyperactivity. Four initial studies (reviewed in (Willcutt, et al., in press), appendix B) appear to support validity of including this designation, and its inclusion as viewed as stimulating further research on this group. However, the more substantial suggestion that restrictive inattentive might be a separate disorder or diagnosis was, after extensive discussion, judged to be inappropriate due to (1) lack of longitudinal data about its differentiation from other presentations over time, (2) preliminary nature of validity data, (3) credibility of DSM if types appear, disappear, reappear across editions without at this point more convincing evidence.

**c. Evidence for the Change**. Longitudinal stability was not present, and this was the main reason for changing from subtypes to presentations. See the summary of the sub-committee literature review (Willcutt, et al., in press). There is initial evidence and clinical intuition for a possible restrictive inattentive group, but there is insufficient longitudinal data to make it a separate disorder or subtype, rather than a presentation, at present.

**d. Potential negative consequences considered**. Two negative consequences were most heavily considered. One would be the loss of a way of organizing heterogeneity in research. However, based on the evidence presented above, the workgroup was confident that this is not a valid way of decreasing such heterogeneity and that such research could continue using the presentations. The second is that clinicians will not code the presentations and will cease to recognize heterogeneity in presentation of ADHD. This concern was deemed to be outweighed by the risk of false reification emerging with the DSM-IV subtypes.

**5) Change the examples in the items**, without changing their content, to accommodate a lifespan relevance of each symptom and to improve clarity.  The committee elected to RETAIN the exact DSM-IV wording of all of the items, but ADD examples that make it easier for clinicians to see the applicability of the criteria across the lifespan.

**a. Magnitude of change. Minor change**

**b. Reason for change.** This was an attempt by the workgroup to respond to the task of rendering all items life span relevant, able to be applicable to preschoolers, children, adolescents, and adults, by creating more versatility in the items by adding examples.

**c. Evidence for change.** Field trials tested revised wording and found no reduction in reliability of the ADHD items.

**d. Negative consequences considered.** It is conceivable that changing the examples available would result in changes in how the items are rated or applied, leading to changes in prevalence or correlates of ADHD. Further, possibly changes in the examples would lead to changes in how published rating scales need to be written. Neither of these potential negative consequences were considered sufficiently likely to over-ride the benefits of more age-applicable examples, in view of the fact that the wording was not changed for the stem and there was no change in reliability in the field trials. However, note that the wording of the examples now in the DSM-5 proposed criteria was changed somewhat from the wording of the examples in the field trials; the wording now in the proposed criteria is CLOSER to DSM-IV than that used in the DSM-5 field trials.

**6) Remove PDD from the exclusion criteria**

**a. Magnitude of change.** Minor change

**b. Reason for change.** There is a growing literature suggesting that ADHD and Autism Spectrum Disorder might co-exist. There is no exclusion of an ADHD diagnosis in the presence of any other developmental disorder or intellectual developmental disorder in DSM-IV. This change is also to bring the ADHD criteria into harmony with the revised criteria for Autism spectrum disorders being proposed by the developmental disorders workgroup.

**c. Evidence for change**. Previous literature in recent years has suggested that: 1) ADHD symptoms occur frequently in Autism Spectrum Disorder (ASD). In some data sets, ADHD is the second most frequent comorbid diagnosis in patients with ASD. There is some evidence for overlapping genetic influences on autistic and ADHD behaviors in community samples. The presence of ADHD symptoms in patients with ASD might confer different neurobiological and clinical correlates from those found in patients with ASD w/o ADHD; 4) Stimulants and other ADHD medication are efficacious in treating ADHD symptoms in patients with ASD (data available includes RCTs).  Thus, it is important to note that the exclusion of ADHD in the presence of ASD defined in the DSM-IV exclude patients with ASD and impairing ADHD symptoms of receiving adequate treatment in countries were reimbursement is based on DSM system. This literature was fully reviewed in a systematic review conducted by the Workgroup in 2010 leading to support for this change.

**d. Negative consequences considered.** Prevalence of ADHD will increase slightly because it is common among children with ASD. ADHD in ASD may be a different disorder than ADHD without PDD. The workgroup did not have the data and resources to adequately evaluate this possibility. However, considerable evidence supports the clinical benefits of allowing co-diagnosis of ASD and ADHD (see above), and the confusing for clinicians that would result from having a conflicting rule out guide in the ASD/PDD versus ADHD criteria was seen as unacceptable. These points outweighed the potential problem of creating an undetected but distinct subgroup of children with ADHD.

**7) Multiple informants:** Modify the pre-amble in criteria A1 and A2 to indicate that information must be obtained from two different informants, preferably a parent and teacher in the case of children and a third part/significant other in cases of adults.

**a. Magnitude of change**: minor change.

**b. Reason for change**. Worldwide guidelines for diagnosing ADHD clearly states that information from multiple reporters should be obtained. This information is already part of the text on ADHD in DSM-IV. However, clinicians tend to not pay sufficient attention to this requirement. This lack of awareness determines an increase in prevalence due to false positive diagnosis of ADHD in cases where symptoms are just noticeable by parents (children and adolescents) or self-report (adults).  The workgroup decided that upload this recommendation as something that should be pursued whenever possible as part of the criteria to deal with this situation.

**c. Evidence for change**. In diagnosing ADHD in children and adolescents, recent investigations have suggested that parents do not provide valid information on child behavior at school (Sayal & Goodman, 2009). For adults, there are doubts whether self-reports of ADHD in childhood are sufficiently reliable.

**d. Potential negative consequences considered.** Obtaining multiple informants is more costly and time consuming for clinicians and could increase costs of clinical diagnosis. For this reason, the workgroup decided to suggest information from different sources as recommendation and not as a requirement for diagnosis.

**8) Still under consideration: For adults, change the threshold of symptoms required.**

 Applegate, B., Lahey, B. B., Hart, E. L., Biederman, J., Hynd, G. W., Barkley, R. A., . . . Shaffer, D. (1997). Validity of the age-of-onset criterion for ADHD: a report from the DSM-IV field trials. *J Am Acad Child Adolesc Psychiatry, 36*(9), 1211-1221.

 Barkley, R. A., & Brown, T. E. (2008). Unrecognized attention-deficit/hyperactivity disorder in adults presenting with other psychiatric disorders. *CNS Spectr, 13*(11), 977-984.

Barkley, R. A., Murphy, K. R., & Fischer, M. (2008). *ADHD in adults : what the science says*. New York: Guilford Press.

Kessler, R. C., Green, J. G., Adler, L. A., Barkley, R. A., Chatterji, S., Faraone, S. V., . . . Van Brunt, D. L. (2010). Structure and diagnosis of adult attention-deficit/hyperactivity disorder: analysis of expanded symptom criteria from the Adult ADHD Clinical Diagnostic Scale. *Arch Gen Psychiatry, 67*(11), 1168-1178. doi: 67/11/1168 [pii]

10.1001/archgenpsychiatry.2010.146

Kieling, C., Kieling, R. R., Rohde, L. A., Frick, P. J., Moffitt, T., Nigg, J. T., . . . Castellanos, F. X. (2010). The age at onset of attention deficit hyperactivity disorder. *Am J Psychiatry, 167*(1), 14-16. doi: 167/1/14 [pii]

10.1176/appi.ajp.2009.09060796

Lahey, B. B., Pelham, W. E., Loney, J., Lee, S. S., & Willcutt, E. (2005). Instability of the DSM-IV Subtypes of ADHD from preschool through elementary school. *Arch Gen Psychiatry, 62*(8), 896-902. doi: 62/8/896 [pii]

10.1001/archpsyc.62.8.896

Mannuzza, S., Castellanos, F. X., Roizen, E. R., Hutchison, J. A., Lashua, E. C., & Klein, R. G. (2011). Impact of the impairment criterion in the diagnosis of adult ADHD: 33-year follow-up study of boys with ADHD. *J Atten Disord, 15*(2), 122-129. doi: 1087054709359907 [pii]

10.1177/1087054709359907

Matte, B., Grevet, E., & Rohde, L. A. (in press). ADHD in adults: a concept in evolution. *Attention-Deficit/ Hyperactivity Disorder*.

 Polanczyk, G., Caspi, A., Houts, R., Kollins, S. H., Rohde, L. A., & Moffitt, T. E. (2010). Implications of extending the ADHD age-of-onset criterion to age 12: results from a prospectively studied birth cohort. *J Am Acad Child Adolesc Psychiatry, 49*(3), 210-216. doi: 00004583-201003000-00004 [pii]

Sayal, K., & Goodman, R. (2009). Do parental reports of child hyperkinetic disorder symptoms at school predict teacher ratings? *Eur Child Adolesc Psychiatry, 18*(6), 336-344. doi: 10.1007/s00787-009-0735-y

Stawicki, J. A., Nigg, J. T., & von Eye, A. (2006). Family psychiatric history evidence on the nosological relations of DSM-IV ADHD combined and inattentive subtypes: new data and meta-analysis. *J Child Psychol Psychiatry, 47*(9), 935-945. doi: JCPP1628 [pii]

10.1111/j.1469-7610.2006.01628.x

Willcutt, E. G., Nigg, J. T., Rohde, L. A., Tannock, R., & Solanto, M. (in press). Meta-analysis of DSM-IV ADHD dimensions and subtypes. *J Ab Psychol*.

**:** If Criterion A1 (Inattention) is met but Criterion A2 (Hyperactivity-Impulsivity) is not met and 3 or more symptoms from Criterion A2 have been present for the past 6 months.

**Inattentive Presentation (Restrictive):** If Criterion A1 (Inattention) is met but no more than 2 symptoms from Criterion A2 (Hyperactivity-Impulsivity) have been present for the past 6 months.

**Predominantly Hyperactive/Impulsive Presentation:** If Criterion A2 (Hyperactivity-Impulsivity) is met and Criterion A1 (Inattention) is not met for the past 6 months.

**Coding note:** For individuals (especially adolescents and adults) who currently have symptoms with impairment that no longer meet full criteria, “In Partial Remission” should be specified.
